# Primary Cardiac Schwannoma: A Meta-Analysis of Individual Case Reports

**DOI:** 10.3390/jcm12103356

**Published:** 2023-05-09

**Authors:** Mohamed Rahouma, Massimo Baudo, Sherif Khairallah, Anas Dabsha, Alessandro Tafuni, Magdy M. El-Sayed Ahmed, Christopher Lau, Erin Iannacone, Yoshifumi Naka, Leonard Girardi, Mario Gaudino, Roberto Lorusso, Stephanie L. Mick

**Affiliations:** 1 Cardiothoracic Surgery Department, Weill Cornell Medicine, New York, NY 10065, USA; 2 Surgical Oncology Department, National Cancer Institute, Cairo University, Cairo 11796, Egypt; 3 Cardiac Surgery Department, Spedali Civili di Brescia, University of Brescia, 25123 Brescia, Italy; 4 Unit of Pathology, Department of Medicine and Surgery, University of Parma, 43126 Parma, Italy; 5 Cardiothoracic Surgery Department, Mayo Clinic, Jacksonville, FL 32224, USA; 6 Department of Surgery, Faculty of Medicine, Zagazig University, Zagazig 44519, Egypt; 7 Department of Cardio-Thoracic Surgery, Maastricht University Medical Centre, Maastricht University, 6202 AZ Maastricht, The Netherlands; 8 Cardiovascular Research Institute Maastricht, 6229 ER Maastricht, The Netherlands

**Keywords:** schwannoma, peripheral nerve sheath tumors, cardiac tumors, malignant, cardiac surgery

## Abstract

Primary cardiac schwannoma (PCS) is a neurogenic tumor that arises from Schwann cells. Malignant schwannoma (MSh) is an aggressive cancer comprising 2% of all sarcomas. Information on the proper management of these tumors is limited. Four databases were searched for case reports/series of PCS. The primary outcome was overall survival (OS). Secondary outcomes included therapeutic strategies and the corresponding outcomes. Among 439 potentially eligible studies, 53 met the inclusion criteria. The patients included had 43.72 ± 17.76 years and 28.3% were males. Over 50% of patients had MSh, with 9.4% also demonstrating metastases. Schwannoma commonly occurs in the atria (66.0%). Left-sided PCS were more common than right-sided ones. Surgery was performed in almost 90% of the cases; chemotherapy and radiotherapy were used in 16.9% and 15.1% of cases, respectively. Compared to benign cases, MSh occurs at a younger age and is commonly located on the left side. OS of the entire cohort at 1 and 3 years were 60.7%, and 54.0%, respectively. Females and males OS were similar up to 2 years follow-up. Surgery was associated with higher OS (*p* < 0.01). Surgery is the primary treatment option for both benign and malignant cases and was the only factor associated with a relative improvement in survival.

## 1. Introduction

Primary cardiac tumors are extremely rare tumors with a prevalence of 0.02–0.056% [1]. Primary cardiac neurogenic tumors are incredibly rare [2,3,4]. These tumors typically arise from Schwann cells and can be either benign or malignant. Malignant peripheral nerve sheath tumors (MPNSTs), also called malignant schwannoma, or neurofibrosarcoma are an aggressive type of cancer that make up only 2% of all sarcomas [5]. They have a high rate of recurrence and metastasis [6,7,8]. Cardiac MPNSTs are even rarer, accounting for only 0.75% of all primary cardiac tumors [2,3,4]. Due to their rarity, there is limited knowledge of the clinical manifestations, imaging features, and proper management of these tumors. Surgical resection is the primary method of treatment for both benign and malignant cases, often followed by radiation and/or chemotherapy to reduce the risk of recurrence and spread in malignant cases [9,10]. Imaging including computed tomography (CT) and magnetic resonance imaging (MRI) can aid in the diagnosis and assessment of the tumor, while transthoracic echocardiography (TTE) is a common method to detect the presence and location of a cardiac mass. It is often difficult to distinguish between benign and malignant cases based on imaging findings, making the perioperative diagnosis a challenging task [11]. Genetic studies have identified specific gene mutations in MPNSTs, such as neurofibromatosis 1 (NF1), polycomb repressive complex 2 (SUZ12), embryonic ectoderm development (EED), the tumor suppressor gene P53 (TP53), and cyclin-dependent kinase inhibitor 2A (CDKN2A), providing a deeper understanding of the complexity of these tumors [12].

In the context of rare diseases, a meta-analysis of case reports (MACRs) can provide valuable information about the diagnosis, management, and outcomes of these conditions, which may not have been studied in large-scale randomized controlled trials (RCTs). MACRs can help to identify patterns and associations among rare cases and can provide insights into the natural history, clinical presentation, and treatment options for rare diseases. They can also help to identify potential risk factors and prognostic indicators for these conditions [13]. Herein, we conducted a MACR for one such rare disease, cardiac schwannoma.

## 2. Materials and Methods

### 2.1. Search Strategy

This systematic review and meta-analysis were conducted according to the preferred reporting items for systematic reviews and meta-analyses (PRISMA) guidelines [14]. The PRISMA flowchart is presented in Supplementary Figure S1. The Ovid MEDLINE, Ovid Embase, Web of Science, and Cochrane Library databases were searched until November 2022 for publications reporting case reports of cardiac schwannomas. The full search strategy is shown in Supplementary Table S1. Furthermore, the references of all studies and reviews were examined to identify additional articles (i.e., “backward snowballing”). To limit publication bias, there were no publication date or language restrictions on the search strategy.

This review was registered with the PROSPERO register of systematic reviews (ID: CRD42023393128). There was no individual patient involvement in this study; as such, research ethics board approval or patient’s consent was not required.

### 2.2. Study Selection

The following steps were taken for study selection: (1) identification of titles of records through database search; (2) removal of duplicates; (3) screening and selection of abstracts; (4) assessment for eligibility through full-text articles; and (5) final inclusion in study. Studies were independently screened for inclusion by two authors (M.B. and A.D.). Discrepancies were arbitrated by a third author (M.R.) to achieve consensus. Articles considered for inclusion were all case reports and case series of cardiac schwannoma.

### 2.3. Data Extraction and Critical Appraisal

Microsoft Office 365 Excel software (Microsoft, Redmond, WA, USA) was used for data extraction. Data on the study period, study center, and country were retrieved. The following patient characteristics were abstracted: age, sex, history of cancer, schwannomatosis, neurofibromatosis, malignancy, position inside the heart, and therapeutic strategy.

The Joanna Briggs Institute critical appraisal tool was used for critical appraisal of the quality of the included case reports [15].

### 2.4. Outcomes of Interest

The primary outcome was to identify the overall survival (OS) of the entire cohort. OS was defined as the length of time from either the date of surgery or the start of treatment of patients diagnosed with PCS that are still alive. Secondary outcomes were the analysis of therapeutic strategies and the corresponding outcomes. Subgroup analyses were performed according to sex, location within the heart, malignancy, and surgery vs. no surgery. Finally, predictors of late mortality were identified.

### 2.5. Statistical Analysis

Continuous data were presented as median and interquartile range and compared using Mann–Whitney U test or as mean and standard deviation and compared using a *t*-test after testing for normality. Categorical data were presented as frequency count and percentages and compared across groups using Chi-square or Fisher’s test, as appropriate.

Overall survival was estimated using Kaplan–Meier methods, presented as survival percentage ± standard error, and compared among groups using a log-rank test. Cox regression was used to identify the predictors of late mortality and was reported as hazard ratio (HRs), and their 95% confidence intervals (95% CI). Firth biased-reduced correction was applied to overcome broad confidence intervals. Variables were selected for multivariate analysis based on their statistical and clinical significance. A barplot was used to assess the geographical trends of reported case reports. Data were analyzed using R version 4.2.2 (R Foundation for Statistical Computing, Vienna, Austria) within RStudio (Vienna, Austria). “Tableone”, “Survival”, and “Survminer” R packages were used.

## 3. Result

### 3.1. Study Selection and Characteristics

The medical literature search identified 439 potentially eligible studies. Seven additional articles were identified through backward snowballing. After the removal of duplicates, 259 studies were screened. Fifty-five full-text articles were assessed for eligibility. Fifty-three papers [16,17,18,19,20,21,22,23,24,25,26,27,28,29,30,31,32,33,34,35,36,37,38,39,40,41,42,43,44,45,46,47,48,49,50,51,52,53,54,55,56,57,58,59,60,61,62,63,64,65,66,67,68] met the inclusion criteria. Supplementary Figure S1. Of note, schwannomas involving the aorta, pulmonary artery, or pericardium were not considered included here. Details of the patient’s characteristics are shown in Table 1 and Supplementary Table S2. The critical appraisal of the included studies is shown in Supplementary Table S3. Most cases were reported in the United States (ten cases), followed by China (nine patients), and Japan (five cases). Supplementary Figure S2.

Only two patients underwent preoperative biopsy to confirm the diagnosis (one malignant, one benign) before surgery was performed. In three patients the diagnosis (all malignant cases) was achieved at autopsy, in two patients because the patient died before the final diagnosis, and in one patient because it was considered terminally ill and the cardiac mass was not further investigated. Two patients did not undergo surgery as the preoperative biopsy showed a malignant histology. In all the other patients the final diagnosis was obtained from the surgical specimen pathologic evaluation.

### 3.2. Meta-Analysis of the Outcomes

The mean age of the overall population was 43.72 ± 17.76 years, with 15 (28.3%) male patients. More than half of the patients presented with a malignant form of the disease, and five (9.4%) with metastases. A total of 46 patients were diagnosed after surgical specimen pathologic evaluation, four patients were diagnosed through a preoperative biopsy and three patients at autopsy. The atrium was the location where the majority of occurrences happened (66.0%), and lesions on the left side were more prevalent than those on the right. Of note, there was only one case of neurofibromatosis and another case of schwannomatosis. There were no embolization events reported. Surgery was performed in almost 90% of the cases with additional chemotherapy and radiotherapy in 16.9% and 15.1% of cases, respectively. The six patients that did not undergo surgery were due to death before diagnosis in two, not amenable to surgery due to extension and/or metastasis in three, or considered terminally ill in one. In four out of these six patients, the tumor was located in the ventricle. Of note, no use of heart transplantation for treatment was reported in any included study. A total of 13 deaths occurred, and seven recurrences of cardiac schwannoma were reported, both of which were in malignant cases. At follow-up, the crude mortality was 26.7% and 26.5% for males and females, respectively (*p* = 0.901), while it was 25.0% and 29.2% for the right and left tumor side, respectively (*p* = 0.999). Outcomes are summarized in Table 1.

Overall survival of the entire cohort at 1, 2, and 3 years were 60.7% ± 9.6%, 54.0% ± 10.6%, and 54.0% ± 10.6%, respectively, Table 2 and Supplementary Figure S3. Malignancy was identified as a risk factor, while surgery was a protective factor for late survival, at both univariable and multivariable Cox regression, Table 3.

### 3.3. Subgroup Analysis

**Figure 1:**
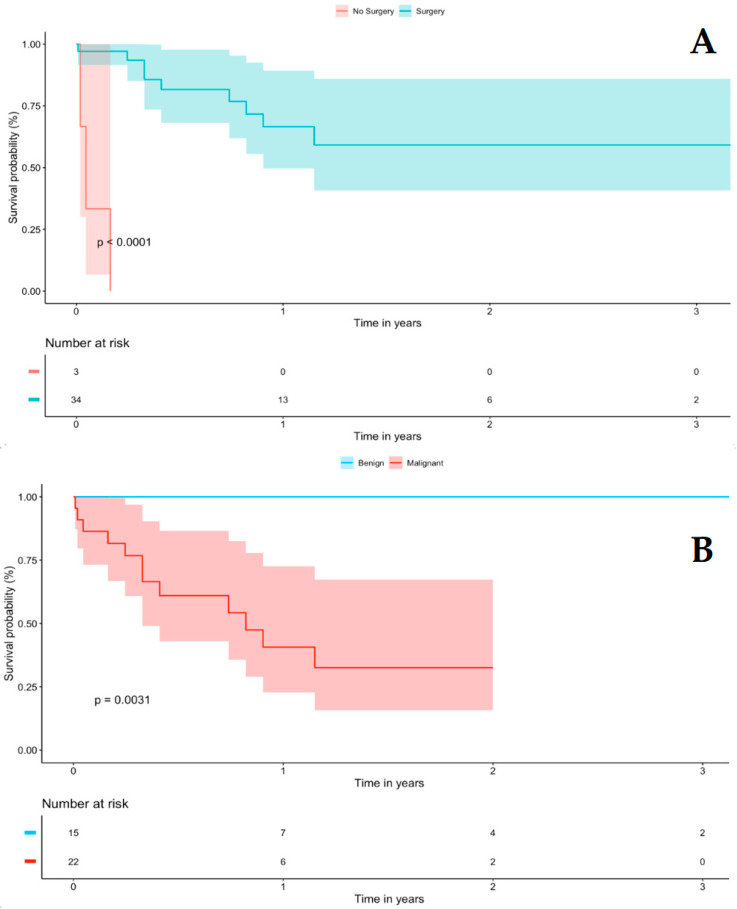
Kaplan–Meier curve of overall survival comparing (**A**) surgery vs. no surgery and (**B**) malignant vs. benign tumors.

No significant differences between sexes were found. Overall, patients undergoing surgery showed higher survival rates compared to no surgery in the whole cohort (log-rank *p* < 0.001) Figure 1A as well as in the malignant subgroup (log-rank *p* = 0.003) Figure 1B, while no survival differences were noted between left and right lesions (log-rank *p* = 0.99) and between sex (log-rank *p* = 0.80) up to 2 years follow-up, Supplementary Figure S4). Only 14 papers reported surgical margins after resection, eight were R0 (four benign, four malignant) and six were R2 (two benign, four malignant). All R0 survived, while three R2 died (all malignant).

Patients with malignant tumors were older (*p* = 0.041), presented with more left-sided lesions (*p* = 0.016), and had worse survival rates (*p* = 0.003). Kaplan–Meier curves confirmed higher mortality rates in the malignant subgroup compared to benign lesions (40.7% ± 12.0% vs. 100% at 1 year, respectively, log-rank *p* = 0.0031) Figure 1B. Patients with malignant disease undergoing surgery had longer survival when compared to no surgery (HR 0.057 [95% CI: 0.0053–0.617, *p* = 0.018] Table 3. Tumor characteristics were not always reported, but among the malignant cases 15/20 (75%) were greater than 5 cm, 4/8 (50%) had a high grading (≥3), and 5/14 (36%) had a high mitotic index (>30%).

Subgroup analysis for late mortality after adjustment for sex revealed the absence of significant difference in relation to prior cancer (*p* = 0.924), laterality (*p* = 0.818), location (*p* = 0.141), and surgery (0.972), Supplementary Figure S5; while after adjustment for cardiac location revealed the absence of significant difference in relation to sex (*p* = 0.818), prior cancer (*p* = 0.942), location (*p* = 0.527), and surgery (0.358), Supplementary Figure S6.

## 4. Discussion

### 4.1. Incidence, Prevalence, and Sites

Primary cardiac tumors are extremely rare tumors [1,69] and cardiac schwannomas are even more rare. The exact incidence and prevalence of these tumors are not well known. These tumors originate from the nerve sheath cells (Schwann cells) of the cardiac plexus, as well as the base maker, and the conduction system. Based on our findings, they are more commonly found in the fifth decade and more common in women than in men (38 vs. 15 cases, respectively). The most common site of origin for cardiac schwannomas is the atria (66.0%), as it is rich in nerves that originate from the superior and inferior cervical sympathetic ganglia 28,59,67. The cardiac branch of the vagus nerve is a common origin. Cardiac schwannomas can also originate from ventricles (28.3%) [42,55,64], and from the conduction system of the heart (septal in location, 3 cases, 5.7%), specifically the atrioventricular (AV) node [57] and the bundle of His. These tumors are less common than those that originate from the atria.

Cardiac schwannoma can be benign or malignant. Benign cardiac schwannomas, also called neurofibromas, are characterized by slow growth and a low likelihood of invading surrounding structures or metastasizing [64]. These tumors can be asymptomatic and discovered incidentally during imaging studies for other reasons [1,26,34,39,42,59,67,70]. The symptoms vary according to size and location (exertional dyspnea, syncope, atrial fibrillation, fatigue, and palpitations) [16,18,19,28,36,50,55,56,57,65,71]. Malignant cardiac schwannomas (also called neurofibrosarcomas, or MPNSTs), on the other hand, are an aggressive subtype. They are characterized by a higher likelihood of invading surrounding structures and metastasizing [22,24]. They are often symptomatic and can cause arrhythmias, embolic events, chest pain, and compression symptoms [17,20,21,22,24,25,26,29,35,43,44,45,46,48,49,51,52,54,60,61,62,63,72]. In this meta-analysis, more than 50% of the reported cases were malignant (29/53 cases) which denotes that the incidence of cardiac MPNST is more than that of benign cases.

Cardiac schwannomas can be sporadic or hereditary. The most well-known genetic disorder associated with familial cardiac schwannomas is neurofibromatosis type 1 (NF1), and type 2 (NF2). Both conditions have an autosomal dominant inheritance that leads to the development of multiple benign tumors, including schwannomas and meningiomas10. Malignant transformation to MPNST can occur on top of atypical plexiform neurofibroma10. Almost half of all MPNSTs arise in the context of NF1 syndrome [9,72,73,74,75], while others occur sporadically or after radiation exposure [7,8]. The most frequent sites of MPNSTs are the extremities (45%), the trunk (34%), and head and neck region (19%) [9,76,77]. Unusual visceral locations of MPNSTs in these contexts have been reported [9,73,78,79]. Studies have suggested that malignant cardiac schwannomas may be more commonly associated with NF1 than with NF2 [10,58]. There is no evidence that spontaneous MPNST is a feature of NF2. King et al. reported that the rate of MPNST per benign cranial nerves schwannoma, particularly the vestibulocochlear nerve schwannoma in NF2 patients is lower than previously reported in the general population [80]. Among 27 cardiac MPNSTs cases included in this meta-analysis, only one case arises on top of preexisting intra-cardiac plexiform neurofibroma [58].

Other genetic disorders associated with familial cardiac schwannomas include schwannomatosis and schwannomatosis-like disorders [67]. Schwannomatosis is an autosomal dominant disorder characterized by the development of multiple schwannomas, and it is caused by mutations in the SMARCB1 and LZTR1 genes [81,82]. Schwannomatosis-like disorders are rare disorders that share clinical and genetic features with schwannomatosis but are caused by mutations in the SMARCE1 gene [83]. In this meta-analysis, we had only one such case in a 46-year-old asymptomatic man [67].

Outside these genetic syndromes, through our cases review, we found that few studies reported another association of cardiac schwannoma with other malignant tumors, such as lung cancer [48,64], renal cell carcinoma [19], ovarian cancer [26,41], breast cancer [51], colon cancer [39], parathyroid carcinoma [20], and lymphoma [72].

### 4.2. Diagnostic Tools

Diagnostic imaging plays an important role in the diagnosis of cardiac schwannomas. It is important to note that the diagnosis is usually confirmed by histological examination. The most used imaging modalities include echocardiography [1], CT [65], MRI [84], and positron emission tomography (PET) [65]. Each of these modalities provides different information (the location, size, and relationship of the tumor to surrounding structures and to differentiate it from other cardiac masses) and can be used in combination to make the diagnosis and plan for treatment. Several studies have shown that the diffusion pattern observed in diffusion-weighted MRI can be utilized to distinguish between benign neurofibroma and MPNST [85,86,87]. According to the findings of Koike H et al, a specific cutoff value of the mean apparent diffusion coefficient (ADC) at 1.85 × 10–3 mm^2^/s resulted in a sensitivity of 80% and a specificity of 74%. Moreover, the mean and minimum ADC values were significantly lower in MPNST than in benign neurofibroma (with *p*-values of 0.03 and 0.003, respectively) [85].

### 4.3. Management

The management of malignant cardiac schwannomas is challenging, and the optimal approach is not well-established due to its rarity. Treatment options are limited, and the best approach is tailored to the individual case and depends on many factors, including the size, location, tumor invasiveness, and the patient’s overall health and treatment goals. Surgery (including heart transplant) is the primary treatment for benign and malignant cardiac schwannomas, and it is usually the first-line treatment, as a general rule for all MPNSTs [8,88]. MPNSTs are well known to be chemo-resistant and relatively radio-resistant [89]. As a result, chemotherapy and radiotherapy are not standard treatments, but they can be considered as salvage therapy in cases of unresectable or recurrent tumors or in cases where surgery is not possible or appropriate, as a neo-adjuvant treatment for locally advanced cases, or in cases with residual disease after resection [90,91,92,93,94,95]. There are limited data on the efficacy of chemoradiation in these cases as studies have reported varying response rates, and the long-term outcomes are not well known. However, chemoradiation therapy might improve the local control but does not affect overall survival [8,96,97]. There is no consensus on the best chemotherapy regimen [92]. Some studies have used a combination of drugs, such as doxorubicin, cisplatin, and etoposide [92,95], while others have used single agents, such as doxorubicin or ifosfamide [91,98,99]. The data about the type of chemotherapy used was not reported in all the included papers of this meta-analysis. However, the most used was ifosfamide among the others (doxorubicin, docetaxel, actinomycin D, vincristine, and cyclophosphamide). Nowadays, with a good molecular understanding of these tumors (which includes upregulation of mitogen-activated protein kinase (RAS/MAPK), phosphoinositide 3-kinase (Pi3K), and the mammalian target of rapamycin (MTOR) pathways [99]), new evolving targeted therapy are now under investigations, and new insights into therapeutic options for MPNST will likely result [100]. The use of radiotherapy for cardiac MPNSTs is limited due to the proximity of the tumor to critical structures and the potential for radiation-induced toxicity that affect the heart, lung, and other structures. Twenty-four out of 27 cases of cardiac MPNSTs in our meta-analysis underwent surgical resection with or without chemoradiation. Only three cases were locally advanced and beyond surgical intervention. All benign cases underwent resection of the tumor with good outcomes.

### 4.4. Prognosis, and Prognostic Factors

Despite aggressive combined modality therapy, 5-year survival rates of MPNSTs range from 35% to 50% in many series [94,101,102]. In our study, surgery was the most important determinant of better survival in both univariate and multivariate analyses. Two-year survival was 59.2% ± 11.2% for cases that underwent surgery vs. zero for the no surgery group. This observation is consistent with several other studies, which report that complete surgical resection is of utmost importance in its impact on survival [94,102]. The crude 5-year actuarial disease-specific mortality rate was 100% for patients who underwent a partial resection, and 5-year survival was 50% for those who underwent complete resection in one series of MPNSTs all over the body [7]. Due to the relatively small sample size, the pathological findings (grade, mitotic index, and tumor size) are not statistically significant predictors of survival in our study. However, most clinical series recognize tumor size as a significant factor impacting survival [94,101,102,103,104]. Zou et al. reported that tumors >10 cm were associated with a three-fold increased risk of developing distant metastases [7]. The mitotic index measured by Ki-67 expression is considered one of the most important prognostic indicators in sarcomas, and elevated Ki-67 expression (i.e., high mitotic index) has been linked to decreased survival in soft tissue MPNSTs [105,106,107]. The mitotic index in our study does not have a negative impact on survival. This might be due to the small sample size, as well as some data missing.

### 4.5. Strength and Limitations

While this meta-analysis of case reports is the first one on cardiac schwannoma, it has several limitations that should be considered. They are based on a small number of cases, often in a diverse group of patients with different characteristics, and may not include all relevant information. They may be subject to publication bias when only surgically resected, positive, or significant results are more likely to be published. This can lead to overestimating the effectiveness of interventions or the frequency of certain conditions.

## 5. Conclusions

In this meta-analysis, more than half of the cases were found to be malignant. Both benign and malignant cases were commonly located in the atria, and there was no significant difference in outcome between males and females. However, malignant cases were found to occur at a younger age and were more frequently located on the left side compared to benign cases. Preoperative diagnosis of these tumors was challenging, with most cases being diagnosed after surgical resection and pathological evaluation. Surgery is the primary treatment option for both benign and malignant cases, while chemotherapy and radiation therapy may be used as adjuvant or neoadjuvant therapy for malignant cases that do not respond well to surgery. For cardiac MPNSTs, survival rates were poor, and on multivariate analysis, complete surgical resection was the only factor that was associated with a relative improvement of 2-year overall survival.

## Figures and Tables

**Table 1:** Patients’ characteristics of the included studies.

Variable	Total (n = 53)	Females (n = 38)	Males (n = 15)	*p*-Value	Benign (n = 24)	Malignant (n = 29)	*p*-Value	Left (n = 27)	Right (n = 23)	*p*-Value
**Age**	43.72 ± 17.76	44.68 ± 15.31	41.27 ± 23.31	0.533	49.17 ± 15.65	39.21 ± 18.39	**0.041**	40.59 ± 16.71	45.35 ± 18.99	0.351
**Male sex**	15 (28.3%)	-	15 (100%)	-	6 (25.0%)	9 (31.0%)	0.858	8 (29.6%)	5 (21.7%)	0.756
**Previous cancer**	10 (19.2%)	6 (16.7%)	4 (26.7%)	0.633	5 (20.8%)	5 (17.9%)	0.999	3 (11.1%)	5 (22.7%)	0.480
**Malignant**	29 (54.7%)	20 (52.6%)	9 (60.0%)	0.858	-	29 (100%)	-	20 (74.1%)	8 (34.8%)	**0.012**
**Metastasis**	5 (9.4%)	3 (7.9%)	2 (13.3%)	0.929	0 (0.0%)	5 (17.2%)	0.096	2 (7.4%)	2 (8.7%)	0.999
**Position**				0.261			0.203			0.256
***Atrium***	35 (66.0%)	25 (65.8%)	10 (66.7%)		18 (75.0%)	17 (58.6%)		17 (63.0%)	16 (69.6%)	
***Ventricle***	15 (28.3%)	12 (31.6%)	3 (20.0%)		6 (25.0%)	9 (31.0%)		7 (25.9%)	7 (30.4%)	
***Valve***	3 (5.7%)	1 (2.6%)	2 (13.3%)		0 (0.0%)	3 (10.3%)		3 (11.1%)	0 (0.0%)	
**Heart side**				0.261			**0.016**			
***Left***	27 (50.9%)	19 (50.0%)	8 (53.3%)		7 (29.2%)	20 (69.0%)				
***Right***	23 (43.4%)	18 (47.4%)	5 (33.3%)		15 (62.5%)	8 (27.6%)				
***Septa***	3 (5.7%)	1 (2.6%)	2 (13.3%)		2 (8.3%)	1 (3.4%)				
**Surgery**	47 (88.7%)	35 (92.1%)	12 (80.0%)	0.440	24 (100%)	23 (79.3%)	0.054	25 (92.6%)	20 (87.0%)	0.850
**Other treatments**				0.572			**<0.001**			0.335
***RTH + CTH***	6 (11.3%)	5 (13.2%)	1 (6.7%)		0 (0.0%)	6 (20.7%)		3 (11.1%)	3 (13.0%)	
***CTH***	3 (3.8%)	1 (2.6%)	2 (13.3%)		0 (0.0%)	2 (6.9%)		1 (3.7%)	0 (0.0%)	
***RTH***	2 (5.7%)	2 (5.3%)	0 (0.0%)		0 (0.0%)	3 (10.3%)		3 (11.1%)	0 (0.0%)	
**Mortality**	13 (27.7%)	9 (26.5%)	4 (26.7%)	0.901	0 (0.0%)	13 (50.0%)	**<0.001**	7 (29.2%)	5 (25.0%)	0.999
***Benign***	0 (0%)	0 (0%)	0 (0%)	0.999	-	-	-	0 (0%)	0 (0%)	0.999
***Malignant***	13 (27.7%)	9 (26.5%)	4 (26.7%)	0.901	-	-	-	7 (29.2%)	5 (25.0%)	0.999
**Recurrence**	7 (15.9%)	6 (18.8%)	1 (8.3%)	0.705	0 (0.0%)	7 (26.9%)	**0.048**	6 (25.0%)	1 (5.9%)	0.237
**Follow-up time (days)**	180 (60, 365)	165 (67.5, 420)	240 (75, 347.5)	0.739	270 (45, 547.5)	165 (90, 356.25)	0.577	180 (90, 365)	285 (42.75, 652.5)	0.548

CTH = chemotherapy; RTH = radiotherapy. Statistically significant *p*-values (*p* < 0.05) are highlighted in bold.

**Table 2:** Kaplan–Meier obtained survival at 1,2, and 3 years.

Survival	1 Year	2 Years	3 Years	Overall Log-Rank *p*-Value
**Overall cohort**				
**Overall**	60.7% ± 9.6%	54.0% ± 10.6%	54.0% ± 10.6%	-
Males *	60.0% ± 19.7%	60.0% ± 19.7%	-	0.800
Females *	59.7% ± 11.3%	52.2% ± 12.1%	52.2% ± 12.1%	
**Benign disease**	100% ± 0%	100% ± 0%	100% ± 0%	**0.003**
**Malignant disease**	40.7% ± 12.0%	35.2% ± 12.1%	-	
**Surgery**	66.6% ± 9.9%	59.2% ± 11.2%	59.2% ± 11.2%	**<0.001**
**No surgery**	NR	NR	NR	
**Malignant subgroup**				
**Males**	47.6% ± 22.5%	47.6% ± 22.5%	-	0.600
**Females**	37.9% ± 14.1%	28.4% ± 13.4%	-	
**Surgery**	47.2% ± 13.3%	37.8% ± 13.6%	-	**<0.001**
**No surgery**	NR	NR	NR	

NR: Not reached. * Landmark analysis was done to evaluate survival differences between males and females after different time cutoffs as follows (A) at 2 years revealed a log-rank *p*-value of 1, then at 1 year revealed a log-rank *p*-value of 0.724, and then at 6 months revealed a log-rank *p*-value of 0.96. Statistically significant *p*-values (*p* < 0.05) are highlighted in bold.

**Table 3:** Factors affecting overall mortality in Cox regression among (A) Overall cohort and (B) Malignant subgroup.

	Univariable Cox *		Multivariable Cox *		Univariable Cox		Multivariable Cox	
Variable	HR (95%CI)	*p*-Value	HR (95%CI)	*p*-Value	HR (95%CI)	*p*-Value	HR (95%CI)	*p*-Value
	Overall Cohort				Malignant Subgroup			
**Age**	1.018 (0.987–1.054)	0.254			1.031 (1.008–1.062)	**0.047**	1.020 (0.988–1.052)	0.207
**Male sex**	0.933 (0.231–2.972)	0.911			0.702 (0.188–2.613)	0.598		
**Previous cancer**	2.439 (0.698–7.437)	0.151			3.553 (0.994–12.69)	0.051		
**Malignant**	18.27 (2.400–2342.3)	**0.001**	14.42 (1.823–1860.7)	**0.006**	–	–		
**Metastasis**	2.170 (0.233–9.241)	0.421			0.814 (0.103–6.408)	0.846		
**Right heart side (ref = left)**	1.015 (0.315–3.063)	0.978			1.606 (0.506–5.087)	0.421		
**Position (ref = atrium)**	Ref.				Ref.			
***Ventricle***	1.667 (0.469–5.260)	0.405			1.715 (0.499–5.889)	0.391		
***Valve***	1.727 (0.181–7.968)	0.567			0.670 (0.081–5.495)	0.709		
**Surgery**	0.027 (0.002–0.171)	**<0.001**	0.046 (0.004–0.298)	**0.002**	0.036 (0.0036–0.3669)	**0.005**	0.057 (0.0053–0.617)	**0.018**

* Firth bias-reduced correction was applied to overcome broad confidence intervals. Statistically significant *p*-values (*p* < 0.05) are highlighted in bold.

## Data Availability

The data that support the findings of this study are available from the corresponding author upon reasonable request.

## Supplementary Materials

The following supporting information can be downloaded at: https://www.mdpi.com/article/10.3390/jcm12103356/s1, Figure S1: PRISMA flow diagram of the included studies; Figure S2: Number of cases per country; Figure S3: Kaplan–Meier curve showing the overall survival of the entire cohort; Figure S4: Kaplan–Meier curve of overall survival comparing (A) females vs. males and (B); Figure S5: Subgroup analysis for late mortality after adjustment for sex; Figure S6: Subgroup analysis for late mortality after adjustment for cardiac location. Table S1: Search strategy; Table S2: Overview of included studies; Table S3: Joanna Briggs Institute Critical Appraisal tool.
