# Supplementary material for: Primary Cardiac Schwannoma: A Meta-Analysis of Individual Case Reports
Source: J Clin Med. 2023 May 9;12(10):3356. doi: 10.3390/jcm12103356 (PMC10219427; doi:10.3390/jcm12103356)

Supplementary Table S1 – Search strategy

Ovid MEDLINE (ALL – 1946 to present)
Searched on November 16, 2022
No language, article type, or publication date restrictions

Line #	Search
1	Neurilemmoma/ and Heart Neoplasms/
2	((cardiac or atrial or artery or arteries or heart or aorta or aortic or pericardial or pericardium) adj3 (schwannoma* or schwann cell* or neurilemmoma* or neurilemoma* or neurinoma* or neurolemmoma or nerve sheath tumor* or nerve sheath tumour*)).tw.
3	1 or 2

Ovid Embase (1974 to present)
Searched on November 16, 2022
No language, article type, or publication date restrictions

Line #	Search
1	neurilemoma/ and heart tumor/
2	((cardiac or atrial or artery or arteries or heart or aorta or aortic or pericardial or pericardium) adj3 (schwannoma* or schwann cell* or neurilemmoma* or neurilemoma* or neurinoma* or neurolemmoma or nerve sheath tumor* or nerve sheath tumour*)).tw.
3	1 or 2

Cochrane Library (Wiley)
Searched on November 16, 2022
No language, article type, or publication date restrictions

ID	Search
#1	((cardiac or atrial or artery or arteries or heart or aorta or aortic or pericardial or pericardium) NEAR/3 (schwannoma or "schwann cell" or neurilemmoma or neurilemoma or neurinoma or neurolemmoma or "nerve sheath tumor" or "nerve sheath tumour")):ti,ab

Web of Science – Core Collection (Clarivate)
Searched on November 16, 2022
No language, article type, or publication date restrictions

ID	Search
#1	TS=((cardiac or atrial or artery or arteries or heart or aorta or aortic or pericardial or pericardium) NEAR/3 (schwannoma or "schwann cell" or neurilemmoma or neurilemoma or neurinoma or neurolemmoma or "nerve sheath tumor" or "nerve sheath tumour"))

Supplementary Table S2 – Overview of the included studies

Author - Year	Journal	Country	Brief case description
**Anderson 2011**	Journal of Cardiac Surgery	USA	Presented with exertional dyspnea, syncope, sever aortic stenosis. Aortic valve replacement and mass resection was performed.
**Arava 2015**	Indian Journal of Pathology and Microbiology	India	Presented with rest dyspnea, palpitations, chest pain, low-grade fever, weight loss, severe tricuspid regurgitation, severe pulmonary hypertension, and mitral valve obstruction. Mass resection was performed.
**Betancourt 1979**	Chest	Puerto Rico	Presented with rest dyspnea, chest pain, and right ventricular outflow tract obstruction. Mass resection was performed.
**Bizzarri 2001**	Journal of Cardiovascular Surgery	Italy	History of renal adenocarcinoma. Presented with dyspnea and chest pain. Mass resection was performed.
**Brandao 2020**	Canadian Journal of Cardiology	Brazil	Previous parathyroidectomy due to a malignant tumor. Presented with palpitations and moderate dyspnea. Surgery not possible for dimension and multiple metastases. Chemotherapy was performed.
**Cho 2012**	Journal of Cardiac Surgery	South Korea	Presented with chest discomfort. Metastasis to lung. Mass resection and lung wedge resection were performed.
**Dammert 1955**	American Heart Journal	Finland	Presented with rest dyspnea, chest pain, cyanosis. Metastatic to mediastinum. Patient died before diagnosis.
**Early 2007**	Journal of Cardiothoracic Surgery	Ireland	Presented with hematemesis. Mass resection was performed.

**Eekhoudt 2019**	Canadian Journal of Cardiology	Canada	Presented with dyspnea, generalized malaise, headache, and night sweats. Surgery not possible for dimension and metastases to lung and brain. Chemo-radiotherapy was performed.
**Eindhoven 2018**	International Journal of Cardiovascular Imaging	The Netherlands	Presented with palpitations, dyspnea and abdominal pain. Partial debulking was performed.
**Factor 1976**	Cancer	USA	History of ovary papillary serous crystadenocarcinoma with liver metastases. Asymptomatic at presentation. Surgery not performed as terminally ill.
**Fan 2012**	American Journal of Clinical Pathology	USA	Presented with generalized swelling. Mass resection was performed.
**Forbes 1994**	Annals of Thoracic Surgery	USA	Presented with atrial fibrillation and chest pain. Mass resection was performed.
**Gelfand 1977**	Journal of Thoracic and Cardiovascular Surgery	Canada	Presented with dyspnea, cyanotic, nausea, epigastric pain, syncope, and tricuspid valve obstruction. Mass resection with chemo-radiotherapy was performed.
**Gleason 1972**	New York State Journal of Medicine	USA	Asymptomatic at presentation. Mass resection was performed.
**Guo 2002**	Chinese Journal of Internal Medicine	China	Presented with dyspnea, chest pain, and mitral valve obstruction. Mass resection and mitral valve replacement was performed.
**Gushmann 1996**	Pathologe	Germany	History of non-hodgkin lymphoma. Presented with dyspnea, mitral and aortic valve insufficiency. Mass resection, mitral valve plasty, aortic valve replacement and chemotherapy were performed.
**Hallman 1966**	Journal of Cardiovascular Surgery	USA	Presented with exertional dyspnea, easy fatigability. Mass resection was performed.

**Hashimoto 1998**	Annals of Thoracic Surgery	Japan	Asymptomatic at presentation. Mass resection was performed.
**Huang 2021**	Chinese Journal of Pathology	China	Presented with cough, exertional dyspnea, and hemoptysis. Mass resection was performed.
**Hwang 2014**	Korean Journal of Thoracic and Cardiovascular Surgery	South Korea	Presented with dyspnea, and chest pain at rest. Mass resection was performed.
**Ivanov 2019**	Russian Journal of Transplantology and Artificial Organs	Russia	Presented with fatigue, and palpitations. Mass resection was performed.
**Jassal 2003**	Journal of Thoracic and Cardiovascular Surgery	Canada	Presented with pleuritic chest discomfort. Mass resection was performed.
**Jung 2015**	Korean Journal of Thoracic and Cardiovascular Surgery	South Korea	History of sigmoid colon cancer. Asymptomatic at presentation. Mass resection was performed.
**Kodama 1995**	Circulation	Japan	Presented with exertional dyspnea and orthopnea. Mass resection was performed.
**Koujanian 2017**	Human Pathology: Case Reports	Canada	History of ovarian cancer. Presented with chest pain, exertional dyspnea, palpitations, and coronary artery disease. Mass resection and coronary artery bypass were performed.
**La Francesca 2007**	Annals of Thoracic Surgery	USA	Asymptomatic at presentation with coronary artery disease. Mass resection and coronary artery bypass were performed.
**Li J 2021**	Journal of Chest Surgery	China	Presented with dyspnea, cough, and upper left back pain. Mass resection was performed.
**Li S 2019**	Medicine	China	Presented with expiratory dyspnea, and edema of face for a month. Surgery aborted as deemed unresectable. Chemo-radiotherapy was performed.

**Li X 2019**	International Journal of Cardiovascular Imaging	China	Presented with cough, expectoration, and dyspnea. Mass resection was performed.
**Machado 2016**	International Journal of Surgical Pathology	Spain	Presented with exertional syncope. Mass resection and radiotherapy were performed.
**Moldovan 2022**	Medicina	Romania	Asymptomatic at presentation. Mass resection was performed.
**Monroe 1984**	Archives of Pathology & Laboratory Medicine	USA	History of metastatic lung adenocarcinoma. Presented with weight loss, exertional chest pain, nail clubbing, and atrial fibrillation. Lung metastasis found inside neurilemmoma. Patient died before diagnosis.
**Morishita 1988**	Clinical Cardiology	Japan	Presented with exertional dyspnea, general fatigue, and fever. Only biopsy was performed.
**Nakamura 2003**	European Journal of Cardio-thoracic Surgery	Japan	Presented with cough and sputum. Mass resection was performed.
**Pauwels 1999**	Histopathology	The Netherlands	History of breast cancer. Presented with severe tricuspid valve regurgitation and right ventricular outflow tract obstruction. Partial debulking was performed.
**Prifti 2014**	Annals of Thoracic Surgery	Albania	Presented with dyspnea, facial swelling, cyanosis, fatigue, tricuspid valve obstruction and superior vena cava compression. Mass resection and chemo-radiotherapy were performed.
**Rausche 2006**	Clinical Research in Cardiology	Germany	Presented with cough, and subfebrile. Mass resection was performed.
**Salem 2011**	European Journal of Internal Medicine	UK	Presented with Exertional shortness of breath, fatigue, worsening exercise tolerance, and mitral valve obstruction. Mass resection and radiotherapy were performed.

**Sevimli 2007**	Echocardiography: A Journal of Cardiovascular Ultrasound and Allied Techniques	Turkey	Presented with palpitations. Partial debulking was performed.
**Sirlak 2003**	Cardiovascular Pathology	Turkey	Presented with dyspnea. Mass resection was performed.
**Son 2015**	Yonsei Medical Journal	South Korea	Presented with palpitations. Mass resection was performed.
**Stepien-Walek 2018**	Kardiologia Polska	Poland	Presented with dyspnea, weakness, significant reduction of exercise tolerance, and mitral valve obstruction. Mass resection was performed.
**Stolf 2006**	Clinics	Brazil	Asymptomatic at presentation. Mass resection was performed.
**Sun 2014**	Cardiovascular Pathology	USA	Presented with generalized swelling, and mitral valve obstruction. Mass resection was performed.
**Ursell 1982**	Human Pathology	USA	Presented with shortness of breath, easy fatigability, ankle edema, increased abdominal girth, and right ventricular outflow tract obstruction. Mass resection was performed.
**Valeviciene 2011**	Gazzetta Medica Italiana	Lithuania	Presented with dyspnea, chest pain, general malaise, and atrial fibrillation. Mass resection and radiotherapy were performed.
**Voluckiene 2012**	Central European Journal of Medicine	Lithuania	Presented with atrial fibrillation. Mass resection and chemo-radiotherapy were performed.
**Wang 2021**	Cardiovascular Pathology	China	Asymptomatic at presentation. Mass resection was performed.
**Wang 2022**	BMC Cardiovascular Disorders	China	History of lung adenocarcinoma. Presented with syncope. Mass resection was performed

**Xiao 2018**	Cardiovascular Flashlight	China	Presented with palpitations, dyspnea, cough, and facial and lower limb oedema. Metastatic at lymph-nodes. Resection not performed as during operation deemed not resectable.
**Yokoyama 2021**	Surgical Case Reports	Japan	History of schwannomatosis. Asymptomatic at presentation. Mass resection was performed.
**Zhu 2019**	Journal of Cardiac Surgery	China	Presented with syncope, chest distress, shortness of breath, accompanied by palpitation, fever, weight loss, and mitral valve obstruction. Partial mass resection, mitral valve replacement, and chemo-radiotherapy were performed.

Supplementary Table S3 – Joanna Briggs Institute Critical Appraisal tool

Paper	Q1	Q2	Q3	Q4	Q5	Q6	Q7	Q8
**Anderson 2011**	Yes	Yes	Yes	Yes	Yes	Yes	Yes	No
**Arava 2015**	Yes	Yes	Yes	Yes	Yes	No	Yes	Yes
**Betancourt 1979**	Yes	Yes	Yes	Yes	Yes	No	Yes	No
**Bizzarri 2001**	Yes	Yes	Yes	Yes	Yes	No	Yes	No
**Brandao 2020**	Yes	Yes	Yes	Yes	Yes	Yes	Yes	Yes
**Cho 2012**	Yes	Yes	Yes	Yes	Yes	Yes	Yes	No
**Dammert 1955**	Yes	Yes	Yes	Yes	Yes	Yes	Yes	Yes
**Early 2007**	Yes	Yes	Yes	Yes	Yes	No	Yes	Yes
**Eekhoudt 2019**	Yes	Yes	Yes	Yes	Yes	No	Yes	No
**Eindhoven 2018**	Yes	Yes	Yes	Yes	Yes	No	Yes	No
**Factor 1976**	Yes	Yes	Yes	Yes	Yes	Yes	Yes	Yes
**Fan 2012**	Yes	Yes	Yes	Yes	Yes	No	Yes	No
**Forbes 1994**	Yes	Yes	Yes	Yes	Yes	Yes	Yes	Yes
**Gelfand 1977**	Yes	Yes	Yes	Yes	Yes	Yes	Yes	No
**Gleason 1972**	Yes	Yes	Yes	Yes	Yes	No	Yes	Yes
**Guo 2002**	Yes	Yes	Yes	Yes	Yes	No	Yes	No
**Gushmann 1996**	Yes	Yes	Yes	Yes	Yes	Yes	Yes	Yes
**Hallman 1966**	Yes	Yes	Yes	Yes	Yes	No	Yes	Yes
**Hashimoto 1998**	Yes	Yes	Yes	Yes	Yes	No	Yes	Yes
**Huang 2021**	Yes	Yes	Yes	Yes	Yes	No	Yes	No
**Hwang 2014**	Yes	Yes	Yes	Yes	Yes	Yes	Yes	Yes
**Ivanov 2019**	Yes	Yes	Yes	Yes	Yes	Yes	Yes	Yes
**Jassal 2003**	Yes	Yes	Yes	Yes	Yes	No	Yes	Yes
**Jung 2015**	Yes	Yes	Yes	Yes	Yes	Yes	Yes	Yes
**Kodama 1995**	Yes	Yes	Yes	Yes	Yes	No	Yes	No
**Koujanian 2017**	Yes	Yes	Yes	Yes	Yes	No	Yes	Yes
**La Francesca 2007**	Yes	Yes	Yes	Yes	Yes	Yes	Yes	Yes
**Li J 2021**	Yes	Yes	Yes	Yes	Yes	No	Yes	Yes
**Li S 2019**	Yes	Yes	Yes	Yes	Yes	No	Yes	Yes
**Li X 2019**	Yes	Yes	Yes	Yes	Yes	No	Yes	Yes
**Machado 2016**	Yes	Yes	Yes	Yes	Yes	No	Yes	Yes
**Moldovan 2022**	Yes	Yes	Yes	Yes	Yes	Yes	Yes	Yes
**Monroe 1984**	Yes	Yes	Yes	Yes	Yes	Yes	Yes	Yes
**Morishita 1988**	Yes	Yes	Yes	Yes	Yes	No	Yes	Yes
**Nakamura 2003**	Yes	Yes	Yes	Yes	Yes	No	Yes	Yes
**Pauwels 1999**	Yes	Yes	Yes	Yes	Yes	No	Yes	Yes
**Prifti 2014**	Yes	Yes	Yes	Yes	Yes	Yes	Yes	Yes
**Rausche 2006**	Yes	Yes	Yes	Yes	Yes	No	Yes	No
**Salem 2011**	Yes	Yes	Yes	Yes	Yes	Yes	Yes	Yes
**Sevimli 2007**	Yes	Yes	Yes	Yes	Yes	No	Yes	Yes
**Sirlak 2003**	Yes	Yes	Yes	Yes	Yes	No	Yes	Yes
**Son 2015**	Yes	Yes	Yes	Yes	Yes	No	Yes	Yes
**Stepien-Walek 2018**	Yes	Yes	Yes	Yes	Yes	Yes	Yes	No
**Stolf 2006**	Yes	Yes	Yes	Yes	Yes	No	Yes	Yes
**Sun 2014**	Yes	Yes	Yes	Yes	Yes	No	Yes	No
**Ursell 1982**	Yes	Yes	Yes	Yes	Yes	Yes	Yes	Yes
**Valeviciene 2011**	Yes	Yes	Yes	Yes	Yes	Yes	Yes	Yes

**Voluckiene 2012**	Yes	Yes	Yes	Yes	Yes	Yes	Yes	Yes
**Wang 2021**	Yes	Yes	Yes	Yes	Yes	No	Yes	Yes
**Wang 2022**	Yes	Yes	Yes	Yes	Yes	Yes	Yes	Yes
**Xiao 2018**	Yes	Yes	Yes	Yes	Yes	No	No	No
**Yokoyama 2021**	Yes	Yes	Yes	Yes	Yes	Yes	Yes	Yes
**Zhu 2019**	Yes	Yes	Yes	Yes	Yes	No	Yes	Yes

Supplementary Figure S1 – PRISMA flow diagram of the included studies

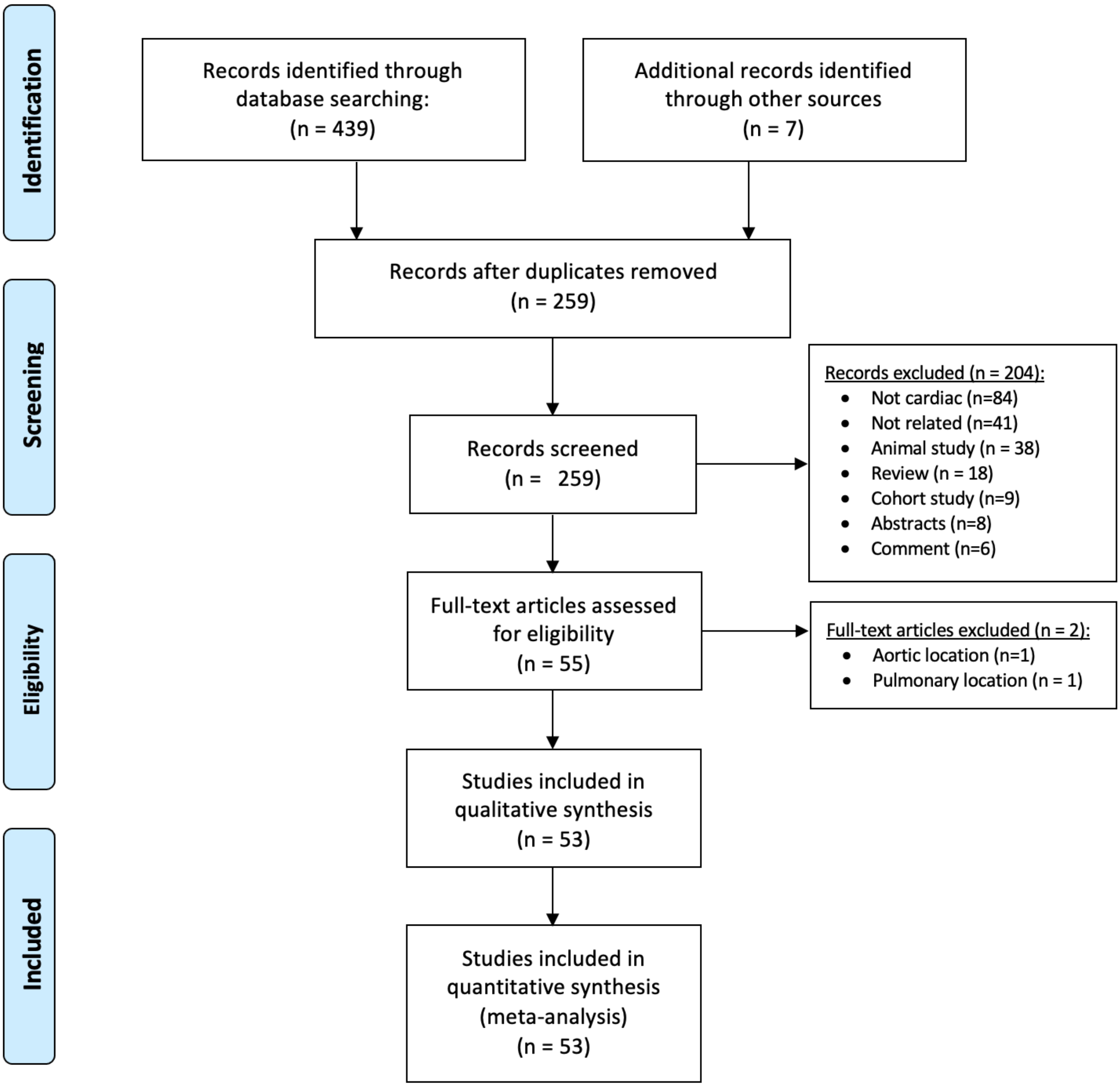

Supplementary Figure S2 – Number of cases per country

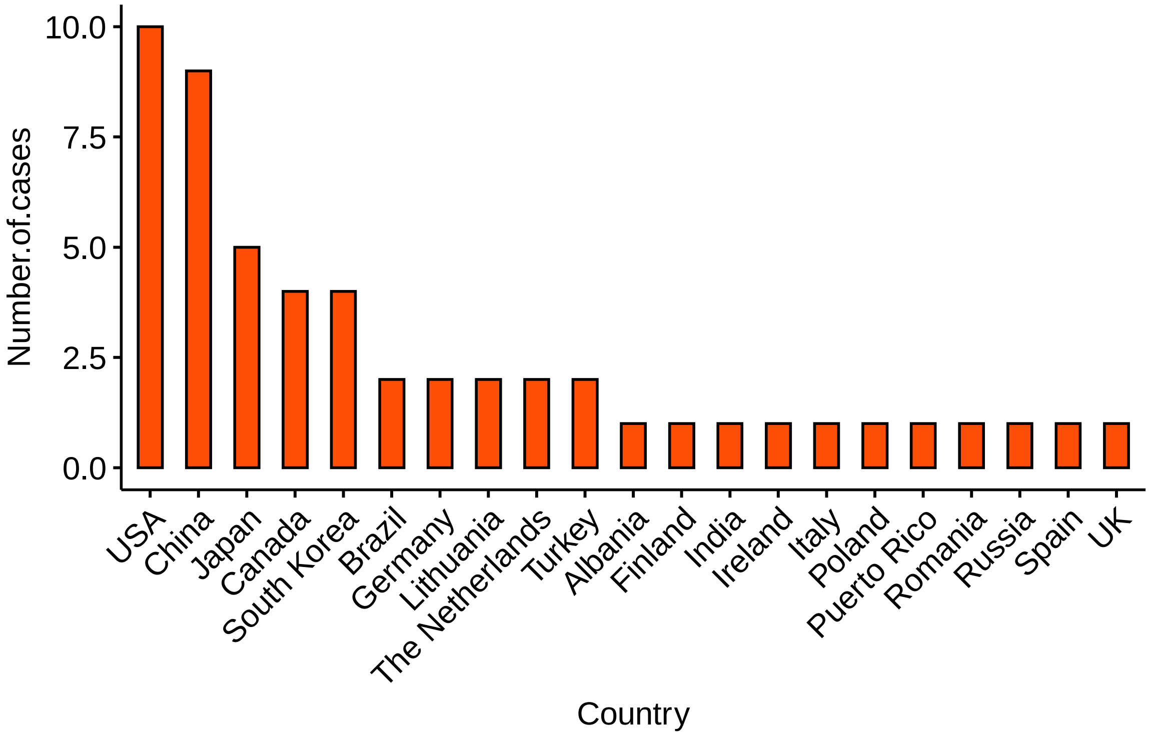

Supplementary Figure S3 – Kaplan-Meier curve showing the overall survival of the entire cohort

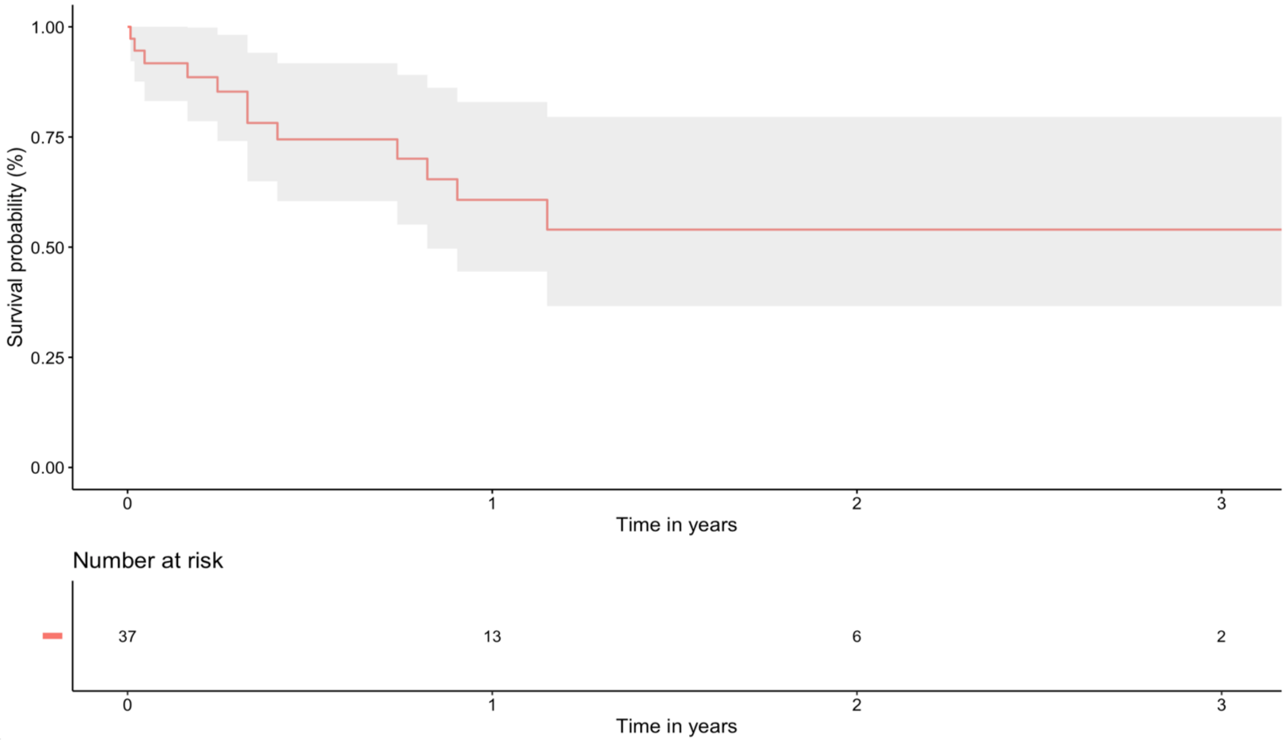

Supplementary Figure S4 – Kaplan Meier curve of overall survival comparing A) females vs males and B) left vs right side of the heart

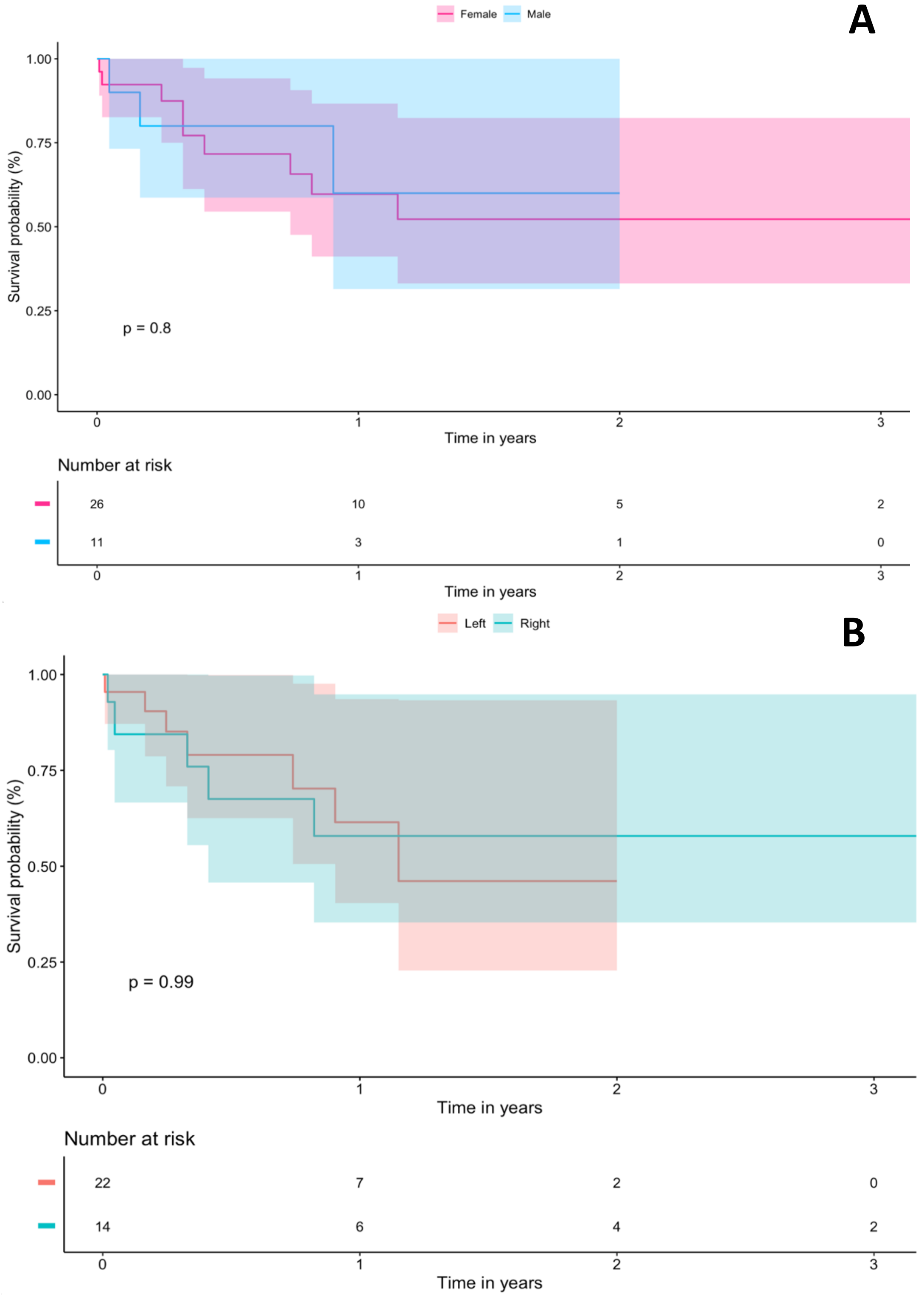

Supplementary Figure S5 - Subgroup analysis for late mortality after adjustment for sex

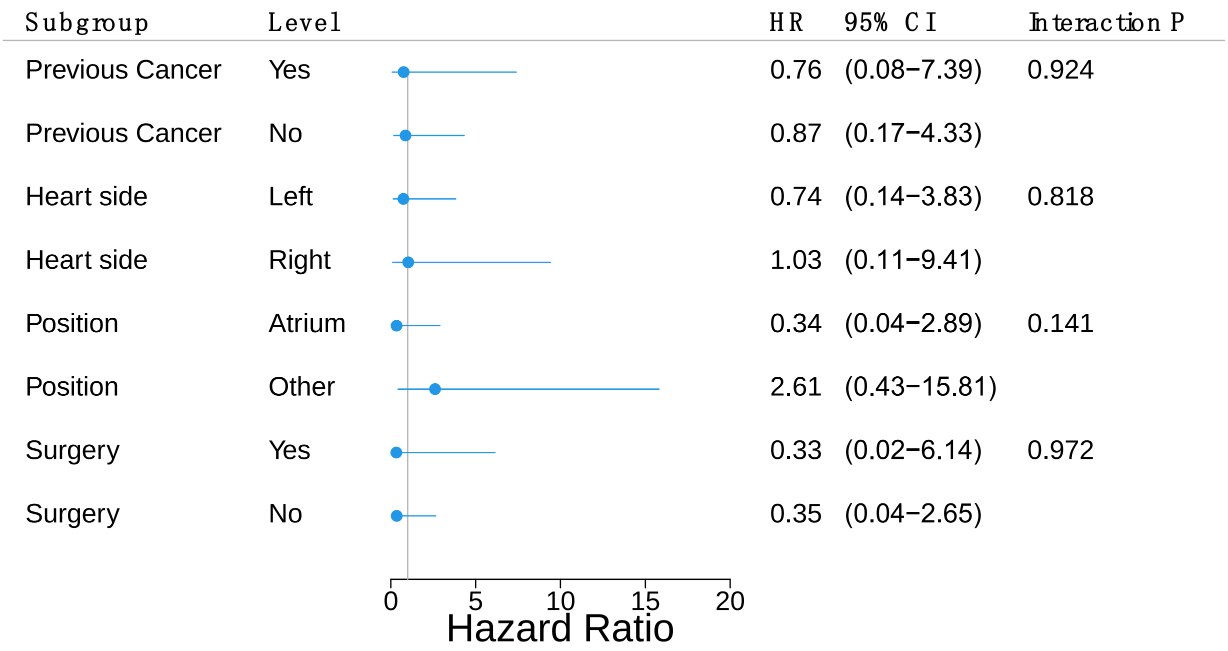

Supplementary Figure S6 - Subgroup analysis for late mortality after adjustment for cardiac location

Subgroup	Level	HR	95% CI	Interaction P
Sex	Male	1.24	(0.11−13.79)	0.818
Sex	Female	0.90	(0.24−3.38)	
Previous Cancer	Yes	0.84	(0.12−6.10)	0.942
Previous Cancer	No	0.92	(0.22−3.88)	
Position	Atrium	0.67	(0.13−3.47)	0.527
Position	Other	1.44	(0.24−8.67)	
Surgery	Yes	0.65	(0.16−2.62)	0.358
Surgery	No	2.64	(0.16−44.22)	

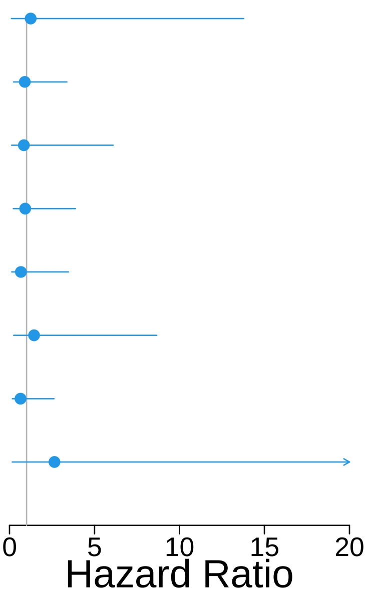

Supplement: Supplementary file 1 [file jcm-12-03356-s001.zip › jcm-2336904-supplementary.pdf]
